# Bisphosphonates Versus Denosumab for Prevention of Pathological Fracture in Advanced Cancers With Bone Metastasis: A Meta-analysis of Randomized Controlled Trials

**DOI:** 10.5435/JAAOSGlobal-D-20-00045

**Published:** 2020-08-01

**Authors:** Humaid Al Farii, Abbey Frazer, Leila Farahdel, Faisal AlFayyadh, Robert Turcotte

**Affiliations:** From the Division of Orthopaedic Surgery, McGill University, Montreal, Quebec, Canada.

## Abstract

Metastasis to the bone is one of the most common complications associated with advanced cancer. Patients with bone metastases are at risk of devastating skeletal related events, including pathological fractures.

**Purpose:**

The aim of this study was to analyze the efficacy of zoledronic acid (ZA) versus denosumab in the prevention of pathological fractures in patients with bone metastases from advanced cancers by evaluating all available randomized controlled trials (RCTs) on this subject.

**Methods:**

A systematic search of electronic databases (PubMed and MEDLINE) was performed to identify all published RCTs comparing ZA with denosumab in prevention of pathological fractures in bone metastases. Risk of bias of the studies was assessed. The primary outcomes evaluated were pathological fractures.

**Results:**

Four RCTs (7,320 patients) were included. Denosumab was superior to ZA in reducing the likelihood of pathological fractures, when all tumor types were combined (odds ratio [OR] 0.86, 95% confidence interval [CI], 0.74 to 0.99, *P* = 0.04). Denosumab was favored, although not statistically significant, over ZA in endodermal origin (breast and prostate) (OR 0.85, 95% CI, 0.68 to 1.05, *P* = 0.13) and mesodermal origin tumors (solid tumors and multiple myeloma) (OR 0.87, 95% CI, 0.71 to 1.06, *P* = 0.16).

**Discussion:**

Denosumab moderately reduces the likelihood of pathological fractures in comparison to ZA in patients with bone metastases with statistical significance. When pathological fractures were grouped by tumor origin (endodermal or mesodermal), no statistical difference was observed between denosumab and ZA. Further long-term studies are needed to confirm the effectiveness of these treatment regimens.

Metastasis to the bone is one of the most common complications associated with advanced cancer. Approximately 350,000 of individuals in the United States die each year with bone metastases, and this figure increases considering patients currently living with bone metastases.^1^ Bone metastases typically stem from malignant breast (73%), prostate (68%), and lung (36%) cancers. Patients with bone metastases are at risk of devastating skeletal related events (SREs), including pathological fractures, requiring prompt referral to an orthopedic surgeon for appropriate management.^2-4^

Bisphosphonates are the main treatment used to reduce the number of SREs in patients with multiple myeloma or bone metastases from advanced cancers (breast, prostate, or solid tumors).^2,5,6^ Zoledronic acid (ZA) is a diphosphonate regarded as one of the current benchmark treatments to reduce, but not completely eliminate, SREs from bone metastases, despite the increased risk of osteonecrosis of the jaw and restrictions in renal insufficiency.^5,7-9^ Therefore, alternate therapies are needed to further reduce the frequency of SREs with fewer adverse effects. Denosumab, a human monoclonal antibody, binds to the receptor activator of nuclear factor kappa-Β ligand (RANKL) and has been shown as a noninferior alternative to ZA.^10^

The aim of our study was to investigate the efficacy of ZA versus denosumab in the prevention of pathological fractures in cancer patients by evaluating randomized controlled trials (RCTs).

## Methods

This meta-analysis was conducted following the Preferred Reporting Items for Systematic Reviews and Meta-Analyses Statement.

We selected RCTs comparing bisphosphonates versus denosumab in patients with bone metastases from advanced cancer and reporting the outcome of pathological fracture prevention. Studies were excluded if they considered children (<16 years) or they had a follow-up of <12 months.

We searched English literature using PubMed and MEDLINE on April 20, 2019, with different terms and synonyms for “bone metastases,” “Bisphosphonates,” and “Denosumab.” In addition, the reference lists of previously published randomized trials and systematic reviews were manually searched for additional eligible studies. The titles and abstracts of the search results were screened, and in case of presumed eligibility, full-text articles were reviewed by two independent reviewers (H.A.F. and A.F.).

Data extraction from each study includes year of publication, randomization method, and patient and treatment characteristics. Seven aspects of the studies related to the risk of bias were assessed, following the instructions in the Cochrane Handbook for Systematic Reviews of Interventions.^11^ The studies were also evaluated specifically for publication bias using a funnel plot.

RevMan software (Version 5.3, The Cochrane Collaboration)^12^ was used for the analysis. Treatment effects were estimated by calculating the odds ratio (OR) with 95% confidence interval (CI) for dichotomous variables and the mean difference with 95% CI for continuous variable. Studies were weighted by the inverse of the variance of the outcome, and a fixed-effects model was used for all analyses.

## Results

**Figure 1:**
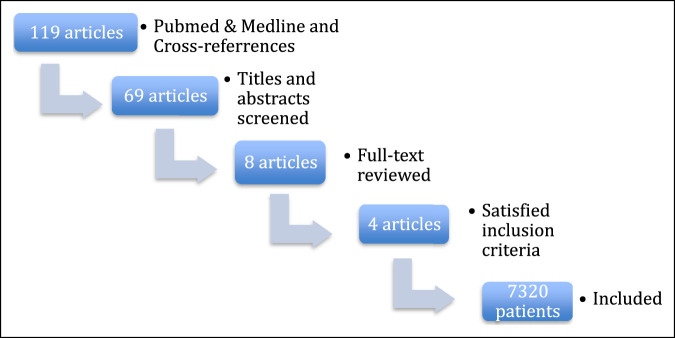
Flow chart illustrating the article screening process.

The search terms, as described above, identified 119 references (Figure 1). Of the eight articles eligible for analysis, four studies needed to be excluded because the numbers of SREs were not listed by type, specifically the number of pathological fractures. Four RCTs were included in the meta-analysis with a total of 7,320 patients (denosumab group = 3,662 and ZA group = 3,658).

### Study Characteristics and Quality

The sample size of the included trials ranged from 797 to 1,026 patients (Table 1). Two trials included patients with mesodermal tumors (or solid tumors and multiple myeloma),^13,16^ and the other two trials included patients with endodermal tumors (breast or prostate cancer).^14,15^ One study only described pathological fractures as lumbar vertebral fractures as part of their first study on SREs.^14^ In addition, one study did not specifically mention the number of pathological fractures, only denoting the total number of SREs.^16^ Amgen was consulted to determine the number of pathological fractures in both the denosumab and ZA groups.

**Table 1:** Characteristics of Studies Included in Meta-analysis

Study	Tumor Type	Study Duration (months)	Groups and Dose	Sample Size (No. of Patients)	No. of Patients Remained by the End of Study	Age (Median)	Sex (Male), n (%)	Pathological Fractures, n (%)
Henry et al^13^	Solid tumor (excluding breast, prostate, multiple myeloma)	34	Denosumab (SC 120 mg Q4W)	800	142	59	531 (66)	92 (12)
			Zoledronic acid (IV 4 mg Q4W)	797	130	61	498 (62)	103 (13)
Stopeck et al^14^	Breast cancer	34	Denosumab (SC 120 mg Q4W)	1026	468	57	8 (0.8)	35^a^ (3.4)
			Zoledronic acid (IV 4 mg Q4W)	1020	461	56	9 (0.9)	56^a^ (5.5)
Fizazi et al^15^	Prostate cancer	41	Denosumab (SC 120 mg Q4W)	950	228	71	950 (100)	137 (14)
			Zoledronic acid (IV 4 mg Q4W)	951	208	71	951 (100)	143 (15)
Henry et al^16^	Solid tumors or multiple myeloma (excluding breast or prostate)	34	Denosumab (SC 120 mg Q4W)	886	180	60	588 (66)	122 (14)
			Zoledronic acid (IV 4 mg Q4W)	890	178	61	552 (62)	139 (16)

a Lumbar vertebral fractures.

In each of the four trials, denosumab was administrated subcutaneously at 120 mg every 4 weeks, and ZA was administered intravenously at 4 mg every 4 weeks. Furthermore, all the four trials concluded that denosumab was moderately more effective in preventing SREs in patients with bone metastases in comparison to ZA with statistical significance.

**Figure 2:**
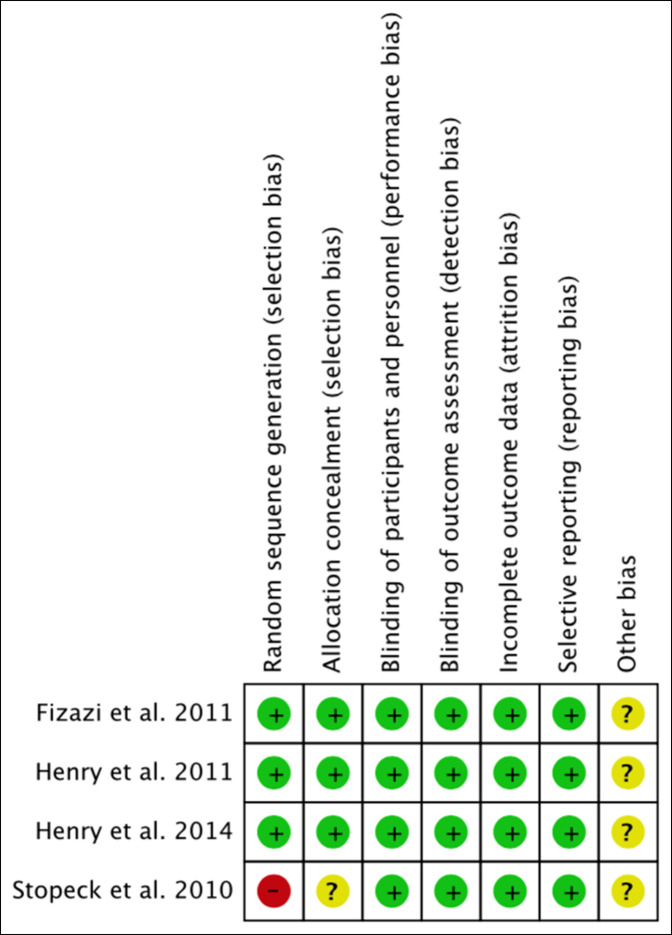
Diagram showing the risk of bias summary according to Cochrane risk of bias assessment tool. “+” = low risk of bias; “−” = high risk of bias; “?” = minimal information and cannot judge risk of bias.

**Figure 3:**
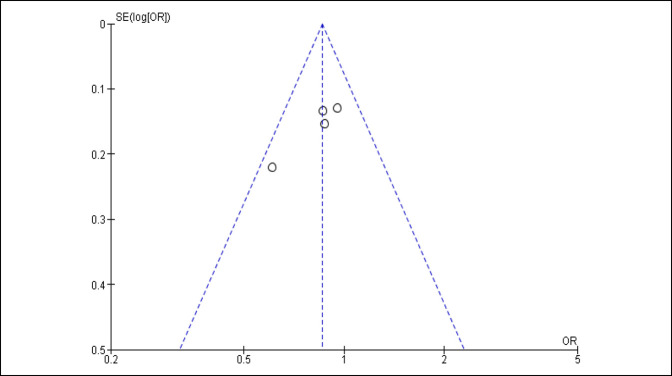
Funnel plot illustrating the level of publication bias. OR = odds ratio, SE = standard estimate.

A summary of the risk of bias in the included studies is presented in Figure 2 along with a funnel plot in Figure 3. The included RCT studies were of moderate to high quality based on the Cochrane bias risk assessment. Only one study did not completely define their approach, leaving randomization and allocation processes unclear.^14^ From the funnel plot, it is shown that the four studies included are well distributed on both sides for publication bias (Figure 3). There is also internal bias contributed by random and systematic bias errors. Random error can be minimized by increasing sample size; however, due to the limited number of research results, random bias is present in the analysis of these studies.

### Effect of Denosumab in Comparison to Zoledronic Acid

**Figure 4:**
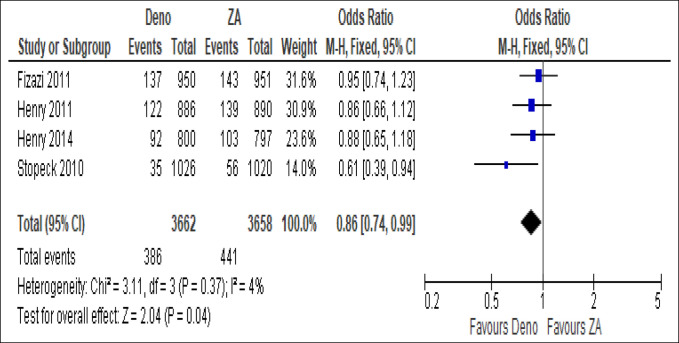
Pathological fracture events presented as odds ratio with 95% confidence interval (CI) for two treatment groups in total after denosumab versus zoledronic acid (ZA) treatment.

**Figure 5:**
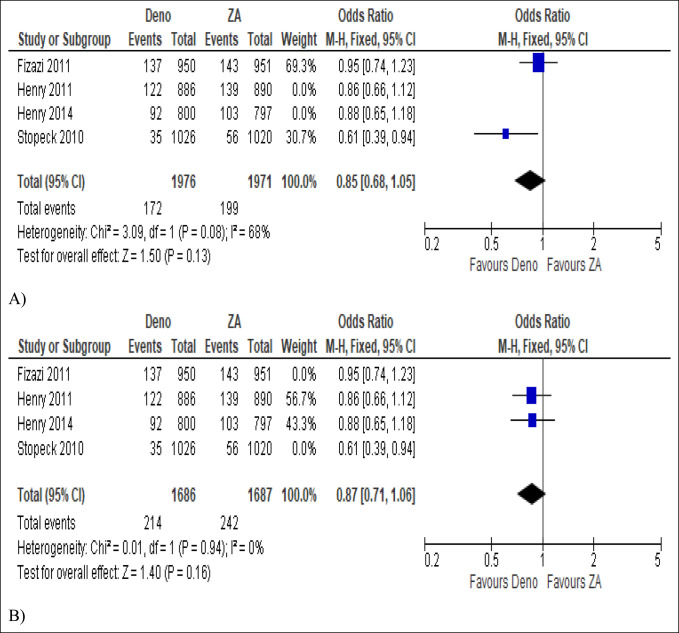
Pathological fracture events presented as odds ratio with 95% confidence interval (CI) for two treatment groups (**A**) in endodermal cancers (breast and prostate) and (**B**) in mesodermal cancers (solid and multiple myeloma) after denosumab versus zoledronic acid (ZA) treatment.

From all studies combined, independent of tumor origin, the effect size estimate favored the denosumab group over ZA in pathological fractures with statistical significance (OR 0.86, 95% CI, 0.74 to 0.99, *P* = 0.04; Figure 4). However, when malignancies were divided by tumor origin, denosumab was not statistically significantly favored over ZA in endodermal origin (breast and prostate) (OR 0.85, 95% CI, 0.68 to 1.05, *P* = 0.13; Figure 5A) and mesodermal origin tumors (solid tumors and multiple myeloma) (OR 0.87, 95% CI, 0.71 to 1.06, *P* = 0.16; Figure 5B).

## Discussion

This meta-analysis of four RCTs that evaluated a total of 7,320 patients shows that denosumab reduces the likelihood of a pathological fracture by 14% in comparison to ZA in the treatment of bone metastases with statistical significance. However, no statistically significant difference was observed between the two groups when patients were categorized by tumor origin, as either endodermal or mesodermal origin. These results run in concordance with previous meta-analyses that concluded denosumab as superior to bisphosphonates, reporting an effect estimate favoring denosumab for SREs, time to first SRE, and incidence of pathological fractures.^17,18^

Based on efficacy alone, denosumab may be an alternative to ZA for advanced tumors to prevent pathological fractures, a major cause of morbidity associated with significant bone pain and disability.^2^ Adverse effects of denosumab and ZA were not evaluated in this study, and thus, we cannot comment on the safety of these respective treatment regimens. The treatments may not be suitable depending on the patients' health conditions.

ZA and denosumab are two antiresorptive treatments with differing mechanisms of action, both helping to reduce the likelihood of SREs, which includes pathological fractures. ZA is a diphosphonate that directly inhibits osteoclastic-mediated activity through accumulation in the mineralized bone matrix and release during bone resorption, especially pathological fractures. Studies have also suggested that ZA may exhibit antitumor effects, including inhibition of tumor cell migration, invasion, proliferation, and viability, further reducing skeletal tumor burden and bone metastasis.^19,20,21,22^ In comparison, denosumab is a monoclonal antibody that binds with high affinity to RANKL, a key mediator in osteoclastic formation and activity, thereby disrupting bone resorption.^23,24^ The disruption of the RANKL signaling pathway by denosumab may explain the enhanced prevention of pathological fractures with denosumab in comparison to ZA. However, the reasoning for which denosumab is more effective than ZA at preventing SREs and pathological fractures in bone metastases remains unclear.

The strength of this meta-analysis was the comprehensive and robust search of the literature. This search ultimately yielded four studies of moderate- to high-quality RCTs based on the Cochrane risk of bias summary. The funnel plot shows that a small number of studies show the symmetrical presence of publication bias; however, it would have been preferred to have included more studies to determine the presence of publication bias with certainty.^25^

Despite the comprehensive search of the literature from electronic databases, the main limitation with this meta-analysis is the small number of included RCTs. Effect estimates of drug treatments in subgroups, categorized by tumor origin, yielded no significant results, with subgroups represented by few RCTs. These effect estimates of drug treatments may vary with more studies and thus, a greater number of patients, included. Furthermore, many others studies have published data comparing denosumab and ZA for SREs; however, the number of SREs by type, such as pathological fractures, was not described.^16,26,27,28,29^ Amgen was consulted for data concerning SREs by type in one study, allowing for inclusion within the analysis.^16^

In conclusion, bisphosphonates are currently used for main treatment to reduce the number of SREs in patients with bone metastases, which however do not come without its own side effects. An alternative, denosumab, was compared with ZA to decrease the risk of SREs. This meta-analysis shows that denosumab is better in reducing the likelihood of pathological fractures compared with ZA in patients with bone metastases. Of note, denosumab was not statistically significant compared with ZA in the reduction of pathological fractures when advanced tumors were grouped by origin. Due to the small number of RCTs included in this study, we cannot recommend the generalized use of denosumab over ZA in the reduction and prevention of pathological fractures in patients with bone metastases. Further large-scale studies are required to confirm the effectiveness of the medications to reduce pathological fractures in advanced cancers.
